# Initial parasitic behaviour of the temporary social parasitic ant *Polyrhachis lamellidens* can be induced by host-like cuticles in laboratory environment

**DOI:** 10.1242/bio.058956

**Published:** 2022-03-24

**Authors:** Yu Kurihara, Hironori Iwai, Nobuaki Kono, Masaru Tomita, Kazuharu Arakawa

**Affiliations:** 1 Systems Biology Program, Graduate School of Media and Governance, Keio University, Fujisawa, Kanagawa 252-0882, Japan; 2 Institute for Advanced Biosciences, Keio University, Tsuruoka, Yamagata, 997-0017, Japan; 3 Faculty of Environment and Information Studies, Keio University, Fujisawa, Kanagawa 252-0882, Japan

**Keywords:** *Polyrhachis lamellidens*, Rubbing behaviour, Host recognition, Chitin, Cuticular compounds

## Abstract

*Polyrhachis lamellidens* is a temporary social parasitic species. When a newly mated queen encounters a host worker, it opens its jaws and then mounts and rubs the body of the host worker, called rubbing behaviour. This behaviour is different from aggressive behaviour and is considered to be a preparatory action before invasion of the host colony. However, it is unclear what cues trigger rubbing behaviour. Therefore, in this study, we used glass beads that imitated the insect body surfaces and searched for triggers. Although *P. lamellidens* did not respond to the cuticular compounds only, cuticular compounds and chitin coatings on glass beads elicited responses that were similar to those towards live samples. The rubbing behaviour of *P. lamellidens* was elicited in response to a cuticle-like surface that mimicked a procuticle by combining the compounds with chitin. These results suggest that host recognition and nest-mate recognition are supported by different mechanisms.

This article has an associated First Person interview with the first author of the paper.

## Abstract (precis)

**Summary:** This study found that initial parasitic behaviour, namely ‘rubbing behaviour’, of the temporary social parasitic species *Polyrhachis lamellidens* can be induced by exposure to a combination of chitin and host cuticular compounds.

## INTRODUCTION

Ants are eusocial insects that generally live in colonies, with genetically related progeny produced by a single queen, and there is communication between individuals (Hölldobler and Wilson, 1990). The existence of a queen is essential for founding a colony. Queens perform a nuptial flight at a specific period and mate with many males. After mating, the queen discards her wings, digs a nest, and lays eggs, thus founding a colony (Hölldobler and Wilson, 1990). Ant species that establish colonies by parasitizing other ant species are called social parasitic species (Buschinger, 2009). Among these species, a temporary parasitic ant usurps the queen by killing her, and the host workers soon function as her own workers (Sakai, 1996). *Polyrhachis lamellidens* (Formicidae: Formicinae), known as a host of myrmecophiles (Iwai et al., 2016), is a temporary social parasitic species that parasitizes *Camponotus japonicus* (Formicidae: Formicinae) (Yano, 1911; Kohriba, 1963 and 1966; Kubota, 1974; Sakai, 1996; Iwai et al., 2021). After the nuptial flight, the newly mated *P. lamellidens* queen locates host workers, mounts them and rubs their entire body. This behaviour is referred to as rubbing behaviour and is considered to be a preparatory action prior to invasion of the host colony (Kohriba, 1963; Kubota, 1974; Sakai, 1996).

Ants communicate with each other via various chemical compounds. Ants are generally hostile to non-nestmates, including other ant species, the same ant species belonging to different colonies, and prey insects. The observation of aggressive behaviour towards glass beads coated with extracts from non-nestmates confirmed that cuticular hydrocarbons induce hostile–aggressive behaviours (Ozaki et al., 2005; Guerrieri et al., 2009). Cuticular hydrocarbons from prey insects also induce hostile behaviour (Liang et al., 2001). Other chemical compounds also known to induce behavioural responses are pheromones. Recruitment pheromone is a guidance pheromone that is laid on a food trail or the trail to the new colony when the colony relocates (Vander Meer and Alonso, 1998). Conversely, alarm pheromone is a volatile pheromone secreted to warn nestmates about an enemy (Vander Meer and Alonso, 1998). According to the above, even though the induced behaviours differ, the behaviours of ants are generally based on the recognition of compounds.

The rubbing behaviour performed by the newly mated *P. lamellidens* queen is distinguishable from aggressive behaviour and has not been observed in other ant species (Kohriba, 1963). Host discrimination is thought to be specific to socially parasitic species and serves to aid in the recognition and parasitization of ant hosts via targeted contacts. *Myrmoxenus ravouxi* (Formicidae: Myrmicinae), which is a social parasitic slave-making ant, shows different attack levels in response to hosts and other species, suggesting that it can distinguish between hosts and non-hosts (Delattre et al., 2013). However, it is not clear whether nestmate-recognition and host-recognition systems are based on the same mechanism. Regarding compounds involved in host recognition, social parasitic bees (genus *Bombus*) rely on substances in the cuticular extracts of the host queen or the host footprint, which is laid at the nest entrance, for host recognition (Cederberg, 1983; Fisher, 1983; Fisher et al., 1993; Bunk et al., 2010; Ayasse and Jarau, 2014). Therefore, in socially parasitic species, the marker used for host recognition is expected to be some kind of compound. However, these markers, including those that induce rubbing behaviour, are still unknown (de la mora et al., 2020).

To identify triggers of *P. lamellidens* rubbing behaviour as a parasitic behaviour rather than an aggressive behaviour, we conducted a bioassay using a glass bead that imitated the body surface of an ant. Identification of the trigger of parasitic behaviour can aid in understanding the host-recognition system. Additionally, new bioassay protocols to induce parasitic behaviour could contribute to rearing and further research.

## RESULTS AND DISCUSSION

### Rubbing behaviour in a laboratory environment

**Fig. 1.**
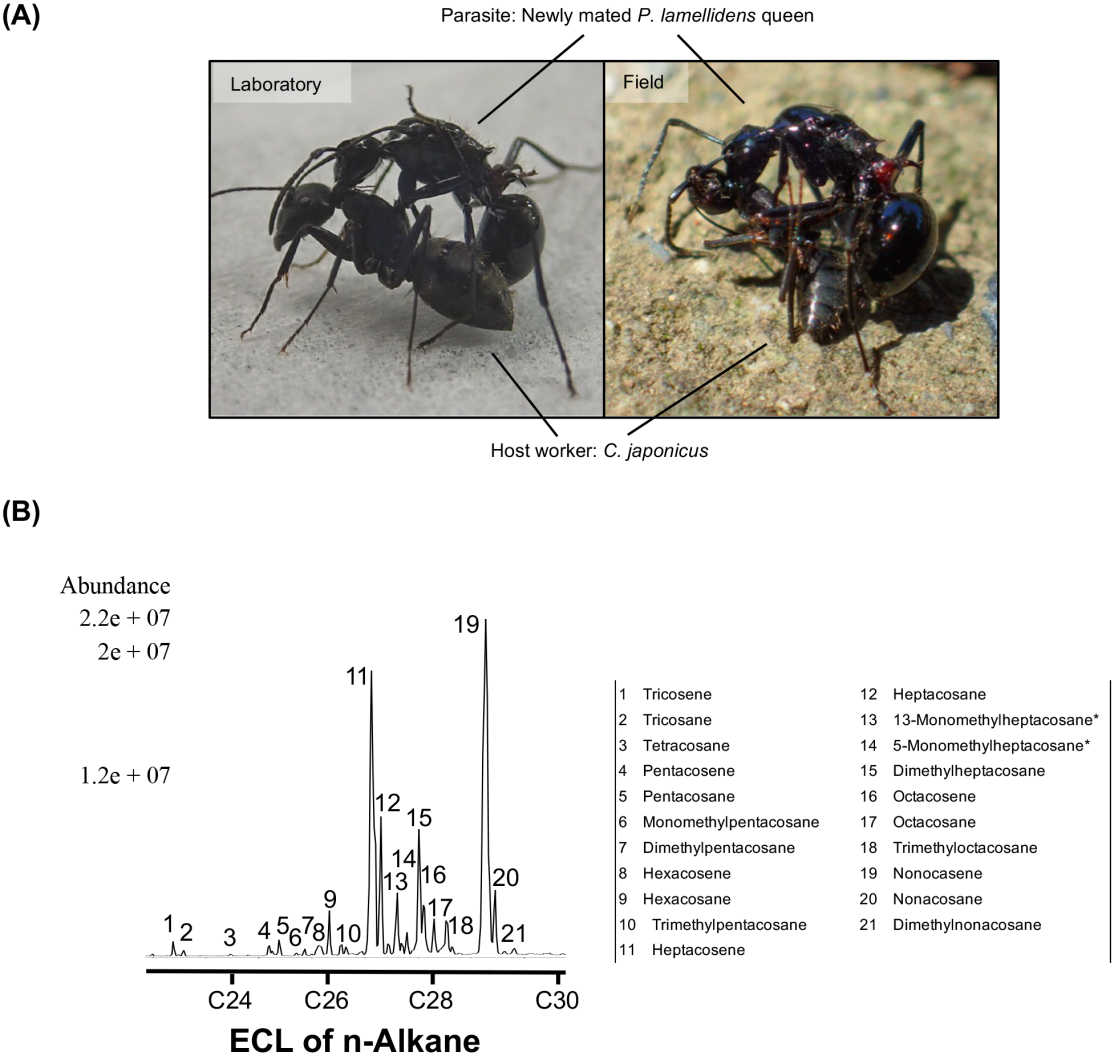
**Rubbing behaviour simulated in the laboratory environment and host cuticular hydrocarbon profile in the CCs measured by GC/MS.** (A) Rubbing behaviour in the laboratory environment (left panel) as well as in the field (right panel). (B) Cuticular hydrocarbon profiles estimated from *C. japonicus* CCs measured by GC/MS. *; the binding site refers to cuticular hydrocarbon profiles from *C. japonicus* workers measured by Ozaki et al. (2005).

We performed a contact test to document the rubbing behaviour of *P. lamellidens* towards *C. japonicus* in the laboratory environment. Prior to contact, the host workers (*C. japonicus*) were cryo-anaesthetized to minimize counterattack against the newly mated *P. lamellidens* queen. As soon as the queen and the worker encountered one other in the arena, the newly mated *P. lamellidens* queen opened her jaws, mounted, and continued to rub the body surface of the cryo-anaesthetized *C. japonicus* worker for approximately 4 min (Fig. 1A). After the *P. lamellidens* queen released the host worker, this behaviour was repeated. Because of the cryo-anaesthesia, the host workers did not resist the *P. lamellidens* queen and did not attempt to flee. After the encounter, the host workers did not appear to be injured and did not die. According to a previous study, this behaviour was characteristic of rubbing behaviour (Kohriba, 1963), and we successfully induced *P. lamellidens* to exhibit rubbing behaviour without counterattack from the host. However, the host workers in the wild are not cryo-anaesthetized; therefore, *P. lamellidens* may select hosts that are easily subjected to rubbing behaviour. In a field experiment involving *Diacamma* sp., the same foragers that were aggressive towards non-nestmates in close vicinity to their nest exhibited non-aggressive behaviours at greater distances from the nest (Uematsu et al., 2019). Furthermore, in *Oecophylla smaragdina*, major workers exhibit a greater degree aggressiveness than minor workers towards non-nestmates (Kamhi et al., 2015). Therefore, in the field, newly mated *P. lamellidens* queens that fortunately contact less aggressive host individuals (far from the nest and/or minor workers) may approach the host colony by repeatedly performing their rubbing behaviour on the host individuals, thus increasing the success rate of parasitism. In this study, the success rate was maximized by cryo-anaesthesia. Limitation of host species is known not only in socially parasitic species but also in various myrmecophiles (Thompson, 1994; Glasier et al., 2018). A high degree of chemical and behavioural specialization is required to break through host defence systems, which is thought to be the reason for host limitation (Thompson, 1994). Furthermore, narrowing of the host range is expected to enable parasitic strategies specific to the restricted host, resulting in more efficient use of the host (Glasier et al., 2018). Host recognition associated with host limitation is also closely related to the success of parasitism. Therefore, the maximization of the success rate of parasitism in the field requires not only host/non-host discrimination but also the selection of individuals vulnerable to parasitism within the host species as mentioned above.

### Analysis of the trigger of rubbing behaviour

**Fig. 2.**
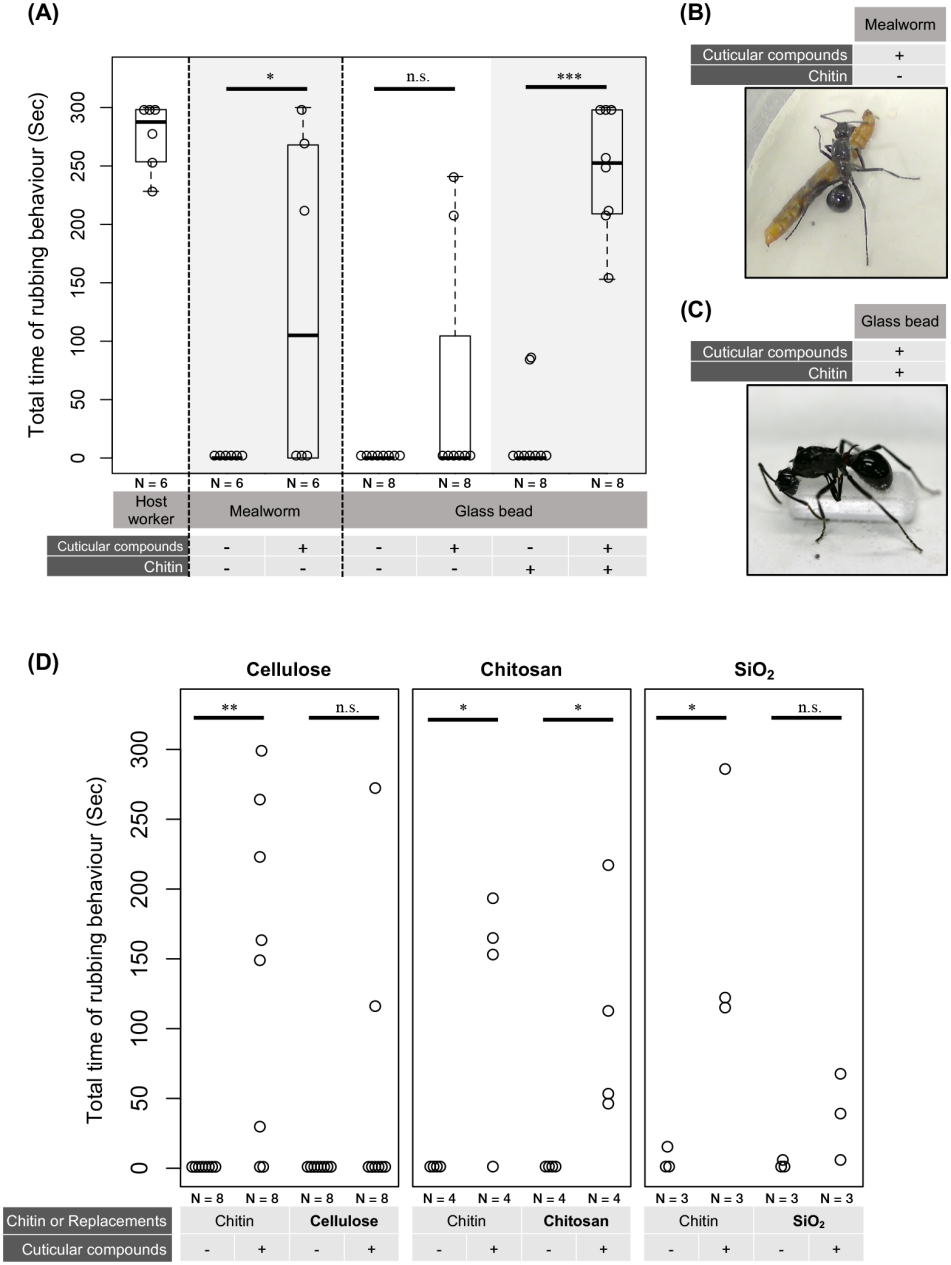
**Rubbing behaviour induced by host cuticular compounds and chitin, or other components.** (A) Total time newly mated *P. lamellidens* queens spent performing rubbing behaviour in each sample. Vertical axis: total amount of time spent performing the rubbing behaviour (Sec). Horizontal axis: samples that had contact with the newly mated *P. lamellidens* queens. The leftmost panel shows a host worker under cryo-anaesthesia. The table shows the rubbing targets (host worker, mealworm, or glass bead) and coating materials (cuticular compounds and/or chitin). The plot shows each sample (one-sided Student's *t*-test; *, significant difference: *P* value<0.05, *n*=6; one-sided Student's *t*-test, ***, significant difference: *P* value<0.0001, *n*=8). (B) Rubbing behaviour towards host CCs applied to mealworms. (C) Rubbing behaviour towards host-CC- and chitin-coated beads. (D) This figure represents the relationship between the kind of chemical component applied to the glass bead and the rubbing behaviour of newly mated *P. lamellidens* queens. The y axis is the total amount of time spent performing the rubbing behaviour (Sec). The x axis indicates the combinations of the chemical components, such as chitin/replacements (cellulose, chitosan or SiO_2_) and cuticular compounds on glass beads. Each chitin sample was prepared as a control (one-sided Student's *t*-test, *, significant difference: *P* value<0.05; one-sided Student's *t*-test, **, significant difference: *P* value<0.01).

To identify the triggers of the rubbing behaviour, we performed a bioassay with a bead. Ants generally rely on cuticular compounds (CCs) for nestmate recognition (Guerrieri et al., 2009), and it has been well established that cuticular hydrocarbons mainly trigger aggressive behaviour (Guerrieri et al., 2009; Ozaki et al., 2005; Sturgis and Gordon, 2012). Therefore, to ensure that the extraction of the CCs was successful, we first checked that the main component, cuticular hydrocarbons, was present. We extracted CCs from a host worker and confirmed that the same cuticular hydrocarbons as previously reported for a *C. japonicus* worker were estimated (Ozaki et al., 2005) (Fig. 1B). To observe the reaction of the *P. lamellidens* queen to host worker CCs, a newly mated *P. lamellidens* queen was confronted with the beads coated with the extracted CCs. Unexpectedly, *P. lamellidens* queens did not perform rubbing behaviour (Fig. 2A). Neither rubbing behaviour nor aggressive behaviour, such as opening the jaws, was performed, and *P. lamellidens* contacted the bead only when climbing over it. To eliminate the effect of the glass surface, we conducted the bioassay with a mealworm instead of a glass bead, as mealworms have a cuticle but not the same CCs as ants. As with the bead, we applied *C. japonicus* CCs to the mealworm surface and exposed the newly mated *P. lamellidens* queen to the mealworm. Half of the *P. lamellidens* queens made significant contact with the mealworm, lasting approximately 4 min (*P*<0.05), and exhibited rubbing behaviour by opening the jaws, rubbing the mealworm, and then rubbing themselves, which was the same behaviour as that observed when the queens were exposed to live host workers (Fig. 2A,B). The difference between the glass bead and mealworm was the surface material. A chitin is widely preserved in arthropod exoskeleton (Merzendorfer, 2006) and it may be a possible explanation for the different responses. Hence, we performed a bioassay using a glass bead coated with a prepared mixture of chitin and *C. japonicus* CCs. Surprisingly, newly mated *P. lamellidens* queens exhibited more frequent rubbing behaviour towards the glass beads coated with the chitin (Fig. 2A,C and Movie 1). The total time spent performing the rubbing behaviour was significantly longer for all the *P. lamellidens* queens (approximately 4 min, *P*<0.0001), and all the queens performed the rubbing behaviour by opening their jaws, rubbing the surface of the bead, and then rubbing their bodies.

*P. lamellidens* exhibited rubbing behaviour towards host worker ants, host CC-coated mealworms, and host CC/chitin-coated glass beads. No rubbing behaviour towards glass beads coated with CCs or only chitin was observed. Therefore, both chitin and host CCs are required for the initiation of rubbing behaviour. One surface-related difference between mealworms and glass beads is that the exoskeleton of mealworms is composed of chitin, a mucopolysaccharide, similar to that in ants. Arthropods have an exoskeleton (cuticle) that protects the body from physical impacts, pathogens, and desiccation (Kramer and Koga, 1986). Chitin is a major component of the cuticles of arthropods, fungi, and nematodes and contributes to the high physical strength of the exoskeleton in arthropods (Merzendorfer, 2006). Since chitin is a component of the cuticle of arthropods, it is supposed that *P. lamellidens* recognizes a target as a host only when chitin is combined with host CCs.

Chitin is a polymer consisting of N-acetylglucosamine (GLcNAc) monomeric units; GLcNAc has various reaction groups, such as the -CH3 methyl group on acetyl groups and -OH groups at the C3 and C6 positions. These groups interact with chemical compounds via anion-cation interactions, chemical or physical adsorption, or electrostatic interaction. Chitin can bind with alkaloids, such as canthin-6 and 4-methoxycanthin-6 (Jaworska et al., 2020). Alkaloids are used as sex pheromones in *Phyllopertha diversa* (Wojtasek and Leal, 1999). A derivative of chitin and diacetylated chitin, chitosan, can also selectively bind with polycyclic aromatic hydrocarbons (Nagai et al., 1999; Jaworska et al., 2020). Moreover, processed chitosan has the potential to bind with various hydrocarbons (Grem et al., 2013). These results suggest that chitin played a role in the recognition of glass beads as insects or ants by *P. lamellidens* or facilitated the adsorption of the CCs to the beads. Regarding the adsorption of CCs, ants release various volatile compounds, and chitin may suppress the volatilization of these compounds. Previous studies using ants have suggested that volatile compounds secreted by the mandibular gland of ants are adsorbed onto the cuticular surface of the whole body (Jaffe, 1987; Hernández et al., 2002). In addition, it has been suggested that ants incorporate external substances into the colony label by adsorbing them onto their cuticular surfaces (Hefetz, 2007). Therefore, chitin is expected to support the misidentification of host CCs or chitin-coated glass beads as host cuticular surfaces by newly mated *P. lamellidens* queens by adsorbing volatile compounds on the host cuticle and suppressing their volatilization after elution.

*P. lamellidens* did not exhibit rubbing behaviour in the absence of CCs. Therefore, host CCs are important in host recognition in *P. lamellidens*. In *Formica japonica*, cuticular hydrocarbons account for 95-98% of CCs, while polar substances account for the remainder (Yamaoka, 1990). Additionally, CCs of *C. japonicus* workers contain various hydrocarbons (Fig. 1B). In ants, cuticular hydrocarbons qualitatively differ among species, and species can be distinguished by these differences (Lenoir et al., 2001). Additionally, different colonies of the same species can have different relative ratios of cuticular hydrocarbons, and ants can discriminate other individuals on the basis of these differences (Lenoir et al., 2001; Ozaki et al., 2005). Therefore, ants likely distinguish between nest mates and non-nest mates by recognizing qualitative (species discrimination) and quantitative (colony discrimination) changes cuticular hydrocarbon profiles (Lenoir et al., 2001). Recent studies have shown that methyl alkanes and alkenes are more important than linear alkanes in recognition (Châline et al., 2005; Martin et al., 2008; Guerrieri et al., 2009; Yusuf et al., 2010). Some hydrocarbons in *C. japonicus* CCs measured in this research may be involved in host recognition. Several studies have suggested that not only cuticular hydrocarbons but also volatile compounds are involved in nestmate recognition (Jaffe and Sánchez, 1984; Hernández et al., 2002; Katzav-Gozansky et al., 2004 and 2008). Furthermore, volatile compounds have been suggested to be adsorbed on the cuticular surface (Jaffe, 1987; Hernández et al., 2002). Therefore, volatile compounds from *C. japonicus* workers can be used as host-recognition markers. On the other hand, since previous studies have also suggested that both volatile and nonvolatile compounds serve as nestmate recognition cues (Akino and Yamaoka, 2012), a combination of compounds with different levels of volatility may be involved in host recognition.

### Replacement of chitin with other compounds

Since chitin and host CCs were found to induce rubbing behaviour, we further tested whether chitin could be replaced by other compounds. When chitin was replaced by chitosan, rubbing behaviour was significantly induced (*P*<0.05) (Fig. 2D). Additionally, a part of *P. lamellidens* performed rubbing behaviour towards beads coated with cellulose or SiO2 powder and host CCs (n.s.) (Fig. 2D). However, all the compounds tended to induce less activity than chitin.

Bioassays using compounds other than chitin have suggested that chitin can be replaced with chitosan, a structural analogue. Chitosan is diacetylated chitin and has a chemical structure very similar to that of chitin. Therefore, the functional group (amine group) shared by chitin and chitosan may interact with host CCs and may have helped retain the CCs on glass beads. However, some degree of rubbing behaviour was also observed towards cellulose- and host-CC-coated beads. Although cellulose does not share functional groups with chitin and chitosan, the rest of the structure and polysaccharide structure are shared among them. Therefore, this common polysaccharide structure may be important. In addition, the powder application may be important due to the generation of static electricity or the increase in the surface area of the bead; these characteristics may result from the slight friction towards the host CC/SiO_2_ powder-coated glass beads. However, none of these alternative compounds seemed to induce as much activity as chitin. Since the insect exoskeleton contains only chitin, chitin may be the most useful compound for mimicking the host surface and actively inducing rubbing behaviour.

Our research supports the hypothesis that newly mated *P. lamellidens* queens identify hosts by recognizing not only the host epicuticle but also the procuticle. Additionally, the chitin may help mimic the insect body surface or verify behaviours in other ant species by suppressing the volatilization of CCs.

## MATERIALS AND METHODS

### Sampling and rearing

Newly mated *P. lamellidens* queens and *C. japonicus* workers were collected in Niigata Prefecture, Japan (October 2018). The ants were identified based on morphological characteristics. *P. lamellidens* queens were reared individually in plastic boxes (5.0×4.5×2.5 cm) with quarter-cut KayDry Wipers (Crecia) moistened with Milli-Q water. These cages were placed in a temperature-controlled incubator at 15°C in the dark. Every week, the plastic box was washed with 70% EtOH, the KayDry wipers were replaced, and food was provided. *C. japonicus* workers were housed in plastic boxes (17.5×8.0×3.0 cm) with plaster spread over the entire floor to maintain humidity (thickness: 5 mm). The boxes were connected to a feeding area and placed in the breeding room, which was maintained at 26°C by an air conditioner, with artificial sunlight conditions (14 h:10 h light:dark) maintained by two TSL-32S (TRUSCO) spiral lights controlled by a PT25 (REVEX) programmable timer. Approximately 5 µl of maple syrup dissolved in Milli-Q water at a ratio of approximately 1:1 was provided every 7-9 days to *P. lamellidens*; 1.5 ml of the same solution was provided to *C. japonicus*. Additionally, frozen mealworms or cockroaches were provided to *C. japonicus*.

### Extraction of CCs

The CC extraction procedures were based on those in a previous study (Akino and Yamaoka, 2012). Ants were cryo-anaesthetized at 4°C for 2 minutes and −20°C for 3 minutes and were placed into a disposable 5 ml glass tube containing 200 µl of hexane for 5 min. After removing the ant from the tube, the CCs eluted in hexane were concentrated by nitrogen gas and then resuspended in 50 µl of hexane.

### Gas chromatography–mass spectrometry (GC–MS) analysis

Extracted CC samples were analysed with GC–MS. GC–MS analysis was performed on an Agilent 6890N GC-5973 MSD system. An HP-5MS column (Agilent, 30 m long, 0.25 mm in diameter, 0.25 µm thick) was used for gas chromatographic separation. The sample injection port temperature was set at 300°C using the splitless mode. Helium carrier gas was set at a flow rate of 0.9 ml/min in constant-flow mode. The oven temperature was set at 40°C for 3 min; increased to 260°C at a rate of 30°C/min, then to 300°C at 15°C/min; and held at 300°C for 18 min. C7 to C40 saturated alkanes were used as standards, and the internal standard was the linear hydrocarbon docosane (C22H46, 10 ng/l µl). GC-MS analysis data were processed using Enhanced ChemStation (Agilent, E02.02.1431).

### Ant contact testing

*C. japonicus* workers were used as host workers for *P. lamellidens*. Before contact with *P. lamellidens*, the host workers were cryo-anaesthetized at 4°C for 2 min and −30°C for 3 min. The contact test was conducted in a plastic case with plaster (76 mm in diameter, approximately 38 mm high). The behaviours were recorded for five minutes (300 s) after newly mated *P. lamellidens* queens encountered *C. japonicus* workers. None of the newly mated *P. lamellidens* queens or *C. japonicus* workers was reused in this or the two following study cases.

### Contact testing with beads

To ensure the inclusion of relatively active ants, *P. lamellidens* queens were selected 4-6 h before the bioassay. Selection was based on the behaviour of the newly mated *P. lamellidens* queen towards the *C. japonicus* worker under cryo-anaesthesia in a plastic case (76 mm in diameter and 38 mm in height). Ant activity was determined by three criteria: (1) the newly mated queen approached the host immediately after contact, (2) the newly mated queen performed rubbing behaviour (not only mounting the host but also rubbing the host's body and applying the body to theirs), and (3) the newly mated queen performed continuous rubbing behaviour for at least 30 s. To prevent contamination or carry over of CCs or cuticular hydrocarbons, mealworms and glass beads were washed with hexane several times before use. The chitin-mixture samples used in the bioassay were prepared by adding 5 mg of chitin powder (Wako) after removal of the solvent by nitrogen gas and redissolution in 80 µl of hexane. Extracted CCs or chitin mixed with CCs were applied using a 100 mm end-to-end tip (AS ONE 3-5998-13) to a mealworm or a trapezoidal glass bead (the application surface was approximately 7 mm long and 2 mm wide). After application, each sample was allowed to stand until the solvent was dry. In the bioassay, the newly mated *P. lamellidens* queen was placed in a plastic arena with plaster (76 mm in diameter, approximately 38 mm high), and the experimental sample was placed in the same arena. Ants recognize various olfactory signals with their antennae (Draft et al., 2018). Therefore, contact between the antennae and samples was considered to be important. An acclimation period of 1 minute was allowed, and a movie was taken for 5 min after first contact of the antenna with the sample; the contact time between *P. lamellidens* and the sample was measured. After 5 minutes, newly mated *P. lamellidens* queens and contact samples were removed from the plastic arena in that order and returned to their breeding case. There was little variation in the time to first contact following the acclimation period (<5 min). Based on the data for total rubbing behaviour time, one-sided Student's *t*-test was performed in R (R core team, 2017) to calculate the amount of time spent performing the rubbing behaviour towards the samples.

### Replacement of chitin with other compounds

To investigate whether some compounds could replace chitin in inducing rubbing behaviour towards a glass bead, we conducted a bioassay using cellulose powder (Wako) or chitosan flakes (Wako) as chemical structural analogues and SiO_2_ powder (Wako) to simulate chitin in a natural form (powder). The chitosan flakes were crushed by a multi-bead shocker (Yasui kikai, 2500 rpm, 30 s for five cycles). Glass beads coated with each compound and host CCs were prepared by the same method used for chitin. The methods for CC elution from host workers and contact tests were the same as above.

## Supplementary Material

Supplementary information
