# Supplementary material for: Initial parasitic behaviour of the temporary social parasitic ant Polyrhachis lamellidens can be induced by host-like cuticles in laboratory environment
Source: Biol Open. 2022 Mar 24;11(3):bio058956. doi: 10.1242/bio.058956 (PMC8966776; doi:10.1242/bio.058956)
Supplement: Supplementary information [file biolopen-11-058956-s1.pdf]

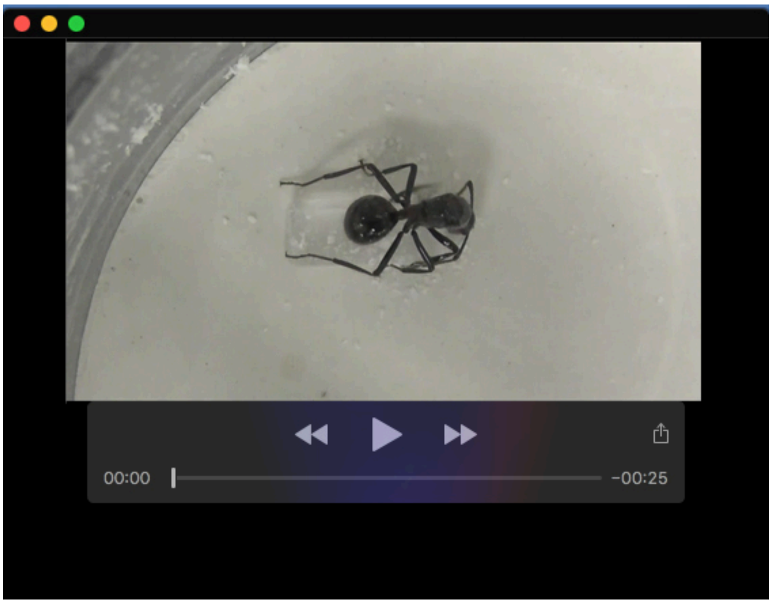

Movie 1. Rubbing behaviour of a newly mated *P. lamellidens* queen towards a glass bead coated with chitin and host CCs.

A newly mated *P. lamellidens* queen and a glass bead coated with chitin and host CCs were placed into a plaster-spread plastic arena. After 1 minute, the movie was taken.

Biology Open • Supplementary information
